# Performance of Edmonton Frail Scale on frailty assessment: its association with multi-dimensional geriatric conditions assessed with specific screening tools

**DOI:** 10.1186/s12877-016-0382-3

**Published:** 2017-01-04

**Authors:** Simone Perna, Matthew D’Arcy Francis, Chiara Bologna, Francesca Moncaglieri, Antonella Riva, Paolo Morazzoni, Pietro Allegrini, Antonio Isu, Beatrice Vigo, Fabio Guerriero, Mariangela Rondanelli

**Affiliations:** 1 Department of Public Health, Experimental and Forensic Medicine, Section of Human Nutrition and Dietetics, University of Pavia, Azienda di Servizi alla Persona di Pavia, Via Emilia 12, Pavia, Italy; 2 Deprtment of Internal Medicine and Medical Therapy, Section of Geriatrics University of Pavia, Azienda di Servizi alla Persona, Pavia, Italy; 3 Research and Development Unit, Indena, Milan Italy

**Keywords:** Edmonton frail scale, Frailty, Functional status, Nutrition, Geriatric assessment

## Abstract

**Background:**

The aim of this study was to evaluate the performance of Edmonton Frail Scale (EFS) on frailty assessment in association with multi-dimensional conditions assessed with specific screening tools and to explore the prevalence of frailty by gender.

**Methods:**

We enrolled 366 hospitalised patients (women\men: 251\115), mean age 81.5 years. The EFS was given to the patients to evaluate their frailty. Then we collected data concerning cognitive status through Mini-Mental State Examination (MMSE), health status (evaluated with the number of diseases), functional independence (Barthel Index and Activities Daily Living; BI, ADL, IADL), use of drugs (counting of drugs taken every day), Mini Nutritional Assessment (MNA), Geriatric Depression Scale (GDS), Skeletal Muscle Index of sarcopenia (SMI), osteoporosis and functionality (Handgrip strength).

**Results:**

According with the EFS, the 19.7% of subjects were classified as non frail, 66.4% as apparently vulnerable and 13.9% with severe frailty.

The EFS scores were associated with cognition (MMSE: β = 0.980; *p* < 0.01), functional independence (ADL: β = −0.512; *p* < 0.00); (IADL: β = −0.338; *p* < 0.01); use of medications (β = 0.110; *p* < 0.01); nutrition (MNA: β = −0.413; *p* < 0.01); mood (GDS: β = −0.324; *p* < 0.01); functional performance (Handgrip: β = −0.114, *p* < 0.01) (BI: β = −0.037; *p* < 0.01), but not with number of comorbidities (β = 0.108; *p* = 0.052). In osteoporotic patients versus not-osteoporotic patients the mean EFS score did not differ between groups (women: *p* = 0.365; men: *p* = 0.088), whereas in Sarcopenic versus not-Sarcopenic patients, there was a significant differences in women: *p* < 0.05.

**Conclusions:**

This study suggests that measuring frailty with EFS is helpful and performance tool for stratifying the state of fragility in a group of institutionalized elderly. As matter of facts the EFS has been shown to be associated with several geriatric conditions such independence, drugs assumption, mood, mental, functional and nutritional status.

## Background

The main characteristics of frailty is a decrease of the reserves in multiple organ systems. The distinction between age and frailty appear to be so blurred that it has been hypothesized that everyone becomes frail when they grow old [1, 2].

In fact, physicians have often used the term frailty to characterize the weakest and most vulnerable subset of older adults. However, ‘frail’ does not mean comorbidity or disability, so this term cannot be chosen to describe the elderly [3].

We can individuate three steps in the frailty process: a pre-frail process, the frailty state and frailty complications [4]. The pre-frail process is clinically silent and the physiological reserves are enough to allow the body to respond adequately to acute diseases, injury, stress or generally any insult with the possibility of complete recovery. The frailty state is characterized by a slow, incomplete recovery after any new acute disease, injury or stress, confirming that the available functional reserves are insufficient to allow a complete recovery. Complications of the frailty process are directly related to physiologic vulnerability resulting from impaired homeostatic reserves and a reduced capacity of the organism to withstand stresses. The risk of falls increases and a functional decline also occurs, leading to disability, poly-medication, an increased risk of hospitalization, cross-infection, institutionalization and death [5–8].

We can conceptualize frailty as a phenotypical state of weight loss, fatigue, and weakness or alternatively as a multidimensional state of vulnerability arising from a complex interplay of biological, cognitive, and social factors [8].

In order to assess frailty in the elderly, Rolfson et al. tested a brief and user-friendly screening interview in both the inpatient and outpatient settings. The “Edmonton Frail Scale”(EFS) was a valid measure of frailty compared to the clinical impression of geriatric specialists after their more comprehensive assessment. The EFS had good construct validity, good reliability and acceptable internal consistency. The interview comprises of 10 areas due to the multi-dimensional presentations of frailty.

The nine domains examined are: cognition, functional performance, general health status, functional independence, social support, pharmacological condition, nutritional aspect, mental condition and continence [9].

The aim of this study was evaluate the performance of Edmonton Frail Scale on frailty assessment, to explore the prevalence of frailty by gender, as assessed by Edmonton Frail Scale, and its association with multi-dimensional conditions assessed with specific screening tools.

## Methods

### Setting

The study was made in Northern Italy. We evaluated white elderly men and women in patients admitted to our physical medicine and rehabilitation division (Santa Margherita Hospital) [10].

Written informed consent was obtained from all participants. In order for the consent to be valid, the patient must had received the necessary information. She/he must had the capacity to consent and the consent must be given voluntarily. Otherwise, when the patient was not able to consent for himself, the consent was gave by the caregiver.

### Study population

Patients with age over 65 years. Subjects not affected by acute illness, severe liver, heart or kidney dysfunction, and with body weight which had been stable for 6 months were included in the study [10]. Data were gathered from the year the beginning of 2011 to the end of 2015.

Prior to commencing the study, we obtained approval from the University of Pavia Research Ethics Board. All participants gave oral consent to participate in this study at the start of the interviews.

### Measurements

#### Assessment of frailty

*Edmonton Frail Scale (EFS):* The EFS assesses nine domains of frailty (cognition, general health status, functional independence, social support, medication usage, nutrition, mood, continence, functional performance) [9, 11].

Test results can be from 0 to 17. The participants were classified conventionally into three categories, and a higher score represents a higher degree of fragility. Severe Frail and non-frail participants were defined according of the EFS score from No frailty (≤5 points) Apparently vulnerable (6 ≤ n ≥ 11 points) and Severe frailty (12 ≤ n ≥ 17) respectively.

Of note, the EFS was validated in the hands of non-specialists who had no formal training in geriatric care and the administration requires few minutes [9].

### Assessment of functional performance

*Handgrip strength test:* Hand grip strength was assessed using a Jamar dynamometer adhering to the standardized protocol recommended by the American Society of Hand Therapists [12]. Handgrip measurement was assessed on the dominant hand and the average value of the handgrip in the two genders was used to define the scores: a score lower than 30 kg for man and lower than 20 kg for women was considered weak [13, 14].

*Barthel Index (BI):* The functional status was assessed by BI score. This scale ranges from 0 (totally dependent) to 100 (totally independent) and assays 10 individual aspects of daily living [15, 16].

### Assessment of cognitive status and mood

*Mini-Mental State Examination (MMSE):* the MMSE is a well-validated and widely used questionnaire to assess global cognitive function, particularly to measure cognitive impairment [17, 18].

*Geriatric Depression Scale (GDS):* The Geriatric Depression Scale was administered to the patients to measure depression. The scale has good internal consistency (α = 0.86) [19], and a sensitivity of ∼ 80% when compared to clinical diagnoses of depression [20, 21].

### Assessment of comorbidities

The number of chronic medical comorbidities and drugs was assessed from the hospital records of each patient.

### Assessment of functional independence

*Activities Daily Living (ADL) and Indipendent Activities Daily Living (IADL):* was measured by interviewing and observing the participants during the interview [22]. Some studies suggest that a composite ADL/IADL scale can be used to represent a single underlying dimension of disability [23, 24].

### Assessment of nutritional status

#### Mini nutritional assessment (MNA)

The MNA is composed of 18 items divided in four categories: anthropometric assessment, general state, dietary assessment and self-assessment. A score ≥ 24 points indicates a good nutritional status. A score from 17 to 23.5 points is an indicator of a risk of malnutrition, while a score ≤ 17 points indicates malnutrition [25, 26].

### Assessment of sarcopenia

Body composition measurement was evaluated using fan‐beam dual‐energy X‐ray absorptiometry (DXA) (Lunar Prodigy DXA; GE Medical Systems, Waukesha, WI, USA). The in vivo coefficients of variation were 4.20% and 0.48% for fat and muscle mass, respectively. The same investigator carried out all measurements for every parameter. Evaluation of “total body” fat mass was obtained by a whole body scan. Skeletal Muscle Index (SMI) is the result of the sum of fat-free soft tissue mass of arms and legs, all divided for height squared [27].

### Assessment of osteoporosis

Bone Mineral Density (BMD) (g/cm2) of the total hip was measured using DXA. BMD was labeled as normal when T- score > 1.0, osteopenic if T-score < −1.0, osteoporosis when T-score ≤ −2.5 [28].

### Assessment of serum albumin

Data of biochemical investigations including serum albumin on the first day of admission were retrieved from hospital records.

### Statistical analysis

All statistical analyses were performed with the SPSS 22.0 package for MAC [27].

Descriptive data are expressed as means ± Standard Deviations of the variables. Mean and standard deviations are reported for continuous variables and percentages for categorical variables.

Linear regression models adjusted for gender and age were calculate for evaluating the association between Edmonton Frail Scale and each evaluation tool with a significant *p*-value <0.05.

A separate one-way ANOVA was performed for assessment differences the EFS score among the three categories of frailty. Comparisons of means for gender were made by using the Student *t*-test for unpaired values (*p* < 0.05).

## Results

### Baseline characteristics

**Fig. 1:**
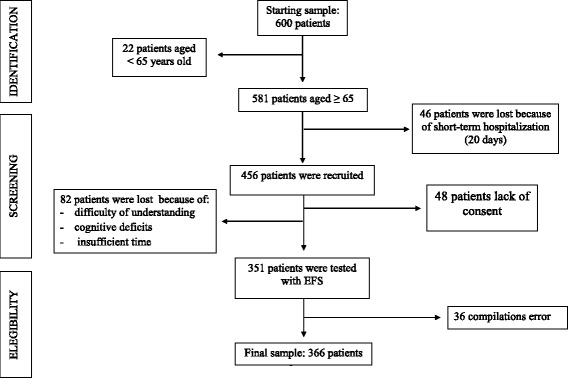
Flow diagram

Figure 1 summarizes patients recruitment. The baseline characteristics for the entire study population (*n* = 366) are listed in Table 1. The study population of 366 participants had a mean age of 81.46 ± 6.55 years. The majority were women (251, 68.6%), and living with an impairment on admission upon hospitalization (ADL: 3.20 points). The mean body mass index (BMI) was slightly overweight (25.05 ± 4.84 kg\m2).

**Table 1:** Baseline characteristics of the sample

Variable	Men (115)	Women (251)	Total (366)	*P*-value
	(Mean ± SD)	(Mean ± SD)	(Mean ± SD)	
Assessment of general data				
Age, years	80.48 ± 6.09	81.90 ± 6.72	81.46 ± 6.55	0.045
Body Mass Index (kg/m^2^)	24.80 ± 4.02	25.17 ± 5.18	25.05 ± 4.84	0.494
EFS, points	8.03 ± 2.93	8.38 ± 2.85	8.27 ± 2.87	0.284
Assessment of functional performance				
Hand Grip DX, kg	20.79 ± 6.08	14.58 ± 5.42	16.51 ± 6.30	*p*<0.001
Bartel Index, points	58.76 ± 25.01	64.09 ± 24.55	62.45 ± 24.77	0.089
Assessment of cognitive status and mood				
MMSE, points	19.32 ± 6.43	18.18 ± 6.14	18.54 ± 6.24	0.192
GDS, points	4.75 ± 3.58	6.18 ± 3.59	5.80 ± 3.58	0.351
Assessment of general health status				
Drugs, n	9.12 ± 3.45	8.41 ± 3.26	8.63 ± 3.33	0.260
Diseases, n	6.24 ± 2.96	5.88 ± 2.71	5.99 ± 2.79	0.063
Assessment of living independence				
ADL, points	3.32 ± 1.88	3.32 ± 1.81	3.20 ± 1.83	0.103
Assessment of sarcopenia				
SMI, n	7.50 ± 1.23	6.41 ± 1.10	6.75 ± 1.25	*p*<0.001
Assessment of osteoporosis				
BMD, g/cm^2^	0.88 ± 0.18	0.72 ± 0.14	0.77 ± 0.11	*p*<0.001
T-SCORE, points	−1.49 ± 1.40	−2.27 ± 1.20	−2.02 ± 1.31	*p*<0.001
Assessment of nutritional status				
Albumin, g/dl	3.67 ± 0.44	3.59 ± 0.57	3.65 ± 0.48	0.224
MNA, points	18.20 ± 3.2	18.06 ± 3.60	18.10 ± 3.47	0.696

Handgrip tests showed a general mean low muscle strength of 16.51 ± 6.30 kg. Mean values of the Barthel Index (62.45 ± 24.77 points) indicated a moderate dependence overall.

The mean values of cognitive status and mood (Mini Mental State Examination: 18.54 ± 6.24 points; Geriatric Depression Scale: 5.80 ± 3.58 points) indicated a state of mild impairment and depression.

In addition, the majority of the patients suffered of osteopenia (hip T-score: −2.02 ± 1.31 SD) and showed a moderate risk of malnutrition (Mini nutritional Assessment: 18.10 ± 3.47 points).

### Total and gender prevalence of frailty with different Edmonton Frail Scale cut-off

As reported in Table 2, the prevalence of frailty according to the level of the Edmonton Frail Scale, 19.7% were classified as non-frail (men: 18.9%; women: 22.4%) subjects, 66.4% as apparently vulnerable (men: 18.5%; women: 47.8%), and 13.9% as severe frail (men: 14.9%; women: 29.8%).

**Table 2:** Distribution of frailty syndrome according to the score on the Edmonton Frail Scale (EFS)

Level of frailty	Men (%)	Women (%)	Total (%)
No frailty (≤5 points)	18.5	22.4	19.7
Apparently vulnerable (6 ≤ n ≥ 11 points)	66.6	47.8	66.4
Severe frailty (12 ≤ n ≥ 17)	14.9	29.8	13.9

### Variables mean among subjects with different frailty statuses

As showed in Table 3, among the three categories of frailty, we found significant differences in mean scores of all screening tools considered (*p* < 0.01). However, there was not found significant differences across the three frailty categories in Albumin level (*p* = 0.079), Skeletal Muscle Index (*p* = 0.194) and BMI (*p* = 0.992).

**Table 3:** Distribution of baseline characteristics among subjects with different frailty categories

	No frailty (≤5 points)	Apparently vulnerable (6 ≤ n ≥ 11 points)	Severe frailty (12 ≤ n ≥ 17)	
	(Mean ± SD)	(Mean ± SD)	(Mean ± SD)	*P* value
Age, years	78.93 ± 7.19	81.80 ± 6.41	82.92 ± 5.47	*p*<0.001
Body Mass Index (kg/m^2^)	24.96 ± 4.17	25.16 ± 5.01	24.85 ± 5.46	0.922
Assessment of functional performance				
Hand Grip DX, kg	19.10 ± 6.43	16.21 ± 6.15	14.12 ± 5.73	*p*<0.05
Bartel Index, points	75,38 ± 21,85	61,6 ± 23,81	51 ± 26,80	*p*<0.001
Assessment of cognitive status and mood				
MMSE, points	20.54 ± 5.86	18.37 ± 6.01	15.1 ± 7.05	*p*<0.001
GDS, points	7.57 ± 3.70	5.52 ± 3.49	4.60 ± 2.61	*p*<0.001
Assessment of general health status				
Drugs, n	7,62 ± 3,42	8,77 ± 3,34	9,38 ± 2,92	*p*<0.05
Diseases, n	5.43 ± 2,42	5,95 ± 2,77	6,92 ± 3,20	*p*<0.05
Assessment of living independence				
ADL, points	4,28 ± 1,69	3,10 ± 1,75	2,25 ± 1,79	*p*<0.001
Assessment of sarcopenia				
SMI, n	6.96 ± 1.22	6.75 ± 1.28	6.48 ± 1.06	0.194
Assessment of osteoporosis				
BMD, g/cm^2^	0.81 ± 0.14	0.77 ± 0.17	0.73 ± 0.16	*p*<0.05
T-SCORE, points	−1.79 ± 0.96	−2.02 ± 1.35	−2.41 ± 1.33	*p*<0.05
Assessment of proteinuria				
Albumin, g/dl	3.73 ± 0.44	3.64 ± 0.50	3.62 ± 0.44	0.079
MNA, points	20,33 ± 3,35	17,96 ± 3,13	15,48 ± 3,11	*p*<0.001

### Association between Edmonton Frail Scale and geriatric evaluation tools

Table 4 Linear regression models adjusted for gender and age showed an association between Edmonton Frail Scale and each specific screening tool.

**Table 4:** Association among EFS with multi-dimensional geriatric conditions assessed with specific screening tools

Edmonton Frail Scale area	Evaluation tools	β	CI 95%	*p*- value
Cognition	Mini Mental State Examination	−0.988	−0.149; −0.048	*p*<0.001
General health status	Number of diseases	0.108	−0.001; 0.217	0.052
Functional independence	Activities daily living (ADL)	−0.512	−0.674; −0.351	*p*<0.001
	Activities daily living (IADL)	−0.338	−0.491; −0.184	*p*<0.001
Social Support	No recorded	-	-	-
Use of medication	Number of drugs	0.110	0.022; 0.199	*p*<0.05
Nutrition	Mini Nutritional Assessment	−0.413	−0.487; −0.338	*p*<0.001
Mood	Geriatric Depression scale	−0.314	−0.332; −0.296;	*p*<0.001
Continence	No recorded	-	-	-
Funcional Performance	Handgrip	−0.114	−0.173; −0.055	*p*<0.001
	Barthel Index	−0.037	−0.049; −0.024	*p*<0.001

Frailty scores assessed by EFS were associated with cognition (MMSE: β = 0.980; *p* > 0.01);); functional independence (ADL: *β* = −0.512; *p* < 0.00); (IADL: β = −0.338; *p* < 0.01); use of medications (β = 0.110; *p* < 0.01); Nutrition (MNA: β = −0.413; *p* < 0.01); mood (GDS: β = −0.324; *p* < 0.01); functional performance (Handgrip: β = −0.114, *p* < 0.01) (Barthel Index: β = −0.037; *p* < 0.01), but not with number of comorbidities (β = 0.108; *p* = 0.052).

### Edmonton score according to sarcopenia and osteoporosis

As showed in Table 5, Between osteoporotic (and not) patients, the mean EFS score did not differ (*p* = 0.365 in women and *p* = 0.088 in men), whereas in Sarcopenic (and not) patients, there was a significant difference in women (*p* < 0.05) but not in men (*p* = 0.318).

**Table 5:** Mean values of EFS score in references to sarcopenia and osteoporosis diagnosis

Sarcopenia			
	Sarcopenic (EFS: Mean ± SD)	No sarcopenic (EFS: Mean ± SD)	*P* value
Women	9.07 ± 2.34	8.24 ± 2.93	*p*<0.05
Men	8.37 ± 3.7	7.73 ± 2.93	0.318
Osteoporosis			
	Osteoporotic (EFS: Mean ± SD)	No osteoporotic (EFS: Mean ± SD)	*P* value
Women	8.50 ± 2.57	8.17 ± 3.00	0.365
Men	9.04 ± 3.32	7.73 ± 2.82	0.088

## Discussion

This study shows the performance of Edmonton Frail Scale on frailty assessment in clinical practice. We demonstrate its association with multi-dimensional geriatric conditions, with specific screening tools, such as MMSE, ADL, IADL, MNA, GDS, Handgrip and Barthel Index.

Moreover, the EFS captures appropriately every single area of frailty. Our study highlights this aspect, showing the association between EFS score and with specific areas, such as independence living, drugs assumption, mood, mental, functional and nutritional status. Moreover we determined relationship between EFS and osteoporosis and sarcopenia. Both these elements are the novelty. To our knowledge other studies have taken in account the relationship between EFS and single areas.

Secondary, this study has taken into account the reliability of EFS to assess the prevalence of frailty in a group of Italian institutionalized elderly patients. The prevalence rate of severe frailty covered the 13.9% of the subjects. The current most cited study was performed by Fried et al. and showed that the prevalence of frailty increased progressively with increasing age, up to a 25% in the age group over 85 years old [5, 8].

Similar progression was reported by Klein et al. in the United States [29]. In the past both the studies of Walston [30] and Graham [31] which considered patients over the age of 65 years, showed a prevalence rate of 6.3% and 7.8% respectively.

In accordance with our study, similar data was reported in the study by Bandeen-Roche, where in a group of elderly patients between 70 and 79 years old, the frailty showed a prevalence of 11.3% [32]. All areas of frailty investigated showed a statistically significant association with the EFS score. If in some ways, finding an association among the EFS and outcome measures is not surprisingly, in our opinion this evidence enlightens the EFS usefulness as a powerful tool (easy to use and less time-expensive) for geriatric multi-dimensional assessment of frailty.

Specifically, the relationship between EFS and MMSE showed a significant association, as described previously in the literature by Kim et al. in [33] and in the study by Faria et al. [34], where the Odds Ratio of developing dementia increases with worsening frailty [33, 34].

The area of frailty, which concerns the general health status was assessed by counting the number of pathologies showing a positive association with the increase of the score of frailty according Edmonton Frail Scale. Our results were in agreement with the results obtained from previous studies in older Americans in which shows a positive relationship between dependence and frailty [35, 36].

Furthermore, drugs were found to be closely associated with the EFS score as already demonstrated in a recent study on an Australian geriatric population [37].

Even the area of the depression, as assessed by the Geriatric Depression Scale (GDS), shows an association with the EFS. In recent study has also highlighted the relationship between an increase of frailty and depression [38].

**Fig. 2:**
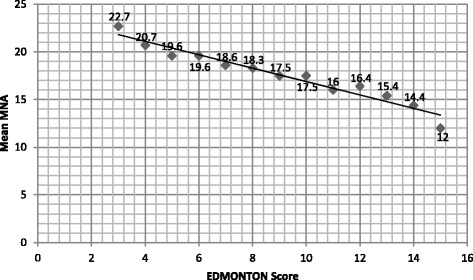
Graphical representation of the average values of MNA for each score of Edmonton Frail Scale

Also the relationship between frailty and nutritional status was investigated. The MNA appears to be associated with the EFS. Among the areas of the frailty, the MNA score was the most associated with EFS, and therefore of potentially considerable of high clinical relevance (as showed in Fig. 2). This point is further justified in the scientific literature in the studies by Izawa et al. [39] which demonstrated that the need for clinical care increases with decreasing nutritional status [39] and by Bollwein et al. [40] in which the percentage of people at risk of malnutrition increased progressively from no frail to severe frailty [40]. Our study also emphasizes the importance of monitoring the state of nutrition in the frail elderly population. In fact, the data show a serious malnutrition both in patients with severe frailty as well as in the apparently vulnerable. Considering the parameters of diagnosis of sarcopenia by gender, related to muscle mass, the mean of the patients enrolled this study were not sarcopenic (mean of Skeletal muscle index in men: 7.51 kg/m2 and mean of Skeletal muscle index in women: 6.41 kg/m2). However, if we consider only muscle strength (through evaluation of the handgrip strength), the preliminary associations within the categories of frailty was significant.

Finally, another aspect of frailty, assessed by the evaluation of bone mineral density (BMD), is osteoporosis. The study considered subjects with average of bone mineral density in a situation of osteopenia. The statistical analysis shows a striking association between T-score and EFS. This finding is confirmed and corroborated if we take into account the association with BMD, which are rather significant. A previous study showed that patients with osteoporosis have a higher risk of developing frailty (OR = 2.1) [41].

A limitation of this study has been the lack of information considering social support and incontinence due to practical considerations in objectively quantifying these parameters for this number of patients. No appropriate scales with defined scores are known. In addition, data were collected only by the administration of the test in the interview with the patient.

Functional performance, measured by a walking test in which measure the time it takes for the patient to get up from a chair, walk about 3 m away and return to sit, were instead represented in the statistical test of Handgrip since this was most easily detectable in immobile patients. Furthermore in patients not able to run the tests mentioned above, the score was awarded after an interview with the caregiver or by evaluating the gait of the patient through the examination by the medical staff.

## Conclusions

In conclusion, this study suggests that EFS is helpful and performance tool to stratifying the state of frailty in a group of institutionalized elderly. Using EFS, we have demonstrated a significant association with all other screening tools that impacting the frail state.

This highlights the potential of this scale as a cheap and convenient performance screening tool to assess frailty upon hospital admission.

## Abbreviations

ADL: Activities daily living
BMD: Bone mineral density
BMI: Body mass index
EFS: Edmonton Frail Scale
GDS: Geriatric depression scale
IADL: Instrumental activities of daily living
MMSE: Mini mental state examination
MNA: Mini nutritional assessment
OR: Ozz ratio
SMI: Skeletal muscle index
